# Safety and Effectiveness of Percutaneous Ethanol Injection as a Treatment for Locally Recurrent Papillary Thyroid Carcinoma

**DOI:** 10.5812/ijem-151696

**Published:** 2024-11-15

**Authors:** Amin Momeni Moghaddam, Mahsa Abbaszadeh, Vajihe Chavoshi, Amir Ebadinejad, Nafiseh Hassanloo, Farhad Hosseinpanah

**Affiliations:** 1 Taleghani Hospital, Shahid Beheshti University of Medical Sciences, Tehran, Iran; 2 Endocrinology and Metabolism Research Center, Imam Khomeini Hospital Complex, Tehran University of Medical Sciences, Tehran, Iran; 3 Department of Endocrinology and Metabolism, Kashan University of Medical Sciences, Kashan, Iran; 4 Department of Surgery, Hartford Hospital, Hartford, CT, 06106, USA; 5 Obesity Research Center, Research Institute for Endocrine Sciences, Shahid Beheshti University of Medical Science, Tehran, Iran

**Keywords:** Percutaneous Ethanol Injection, Papillary Thyroid Carcinoma, Thyroid Nodule

## Abstract

**Background:**

Reoperation for recurrent papillary thyroid cancer (PTC) is associated with a high risk of complications and limited success in achieving sustained remission. Percutaneous ethanol injection (PEI) presents a potential non-surgical alternative for managing locally recurrent PTC.

**Objectives:**

This study aimed to evaluate the safety and effectiveness of PEI in treating recurrent PTC.

**Methods:**

From October 2017 to September 2021, PEI was administered to 39 recurrent lesions (23 lateral and 16 central) in 17 patients with PTC. The median follow-up duration was 21.4 months (range, 4.1 - 37.9), with ethanol injections delivered every 3 months under ultrasound (US) guidance as needed.

**Results:**

Most patients tolerated the treatment well, experiencing only mild local pain, though one patient reported Horner syndrome following the procedure. In terms of treatment frequency, 31 lesions required 3 or fewer injections, while the remaining lesions required more. The mean initial volume of the lesions decreased from 0.12 mm³ (range: 0.06 - 0.34 mm³) to 0.03 mm³ (range: 0.0 - 0.14 mm³), representing an average reduction of 72.6% (range: 20.0 - 100.0%). Of the 39 lymph nodes treated in 17 patients, 21 lymph nodes (54%) were completely resolved. Seven lymph nodes remain under ongoing ethanol treatment, while 11 lymph nodes in 4 patients were addressed with alternative treatments, including surgery.

**Conclusions:**

Percutaneous ethanol injection appears to be a safe and effective treatment option for managing locally recurrent thyroid carcinomas in select patients. However, further comparative, prospective, long-term studies are needed to evaluate PEI’s impact on patient survival and recurrence rates.

## 1. Background

Papillary thyroid carcinoma (PTC), accounting for approximately 80% of thyroid cancers (1), is a well-differentiated, slow-growing tumor that carries a high risk of locoregional lymph node metastasis and recurrence (2). According to the American Thyroid Association (ATA) criteria, the risk of structural recurrence ranges from 3 - 13% in low-risk patients, 21 - 36% in intermediate-risk patients, and around 68% in high-risk patients (3). Recurrence most commonly occurs in the neck lymph nodes following initial PTC treatment (4). Standard management of recurrent PTC typically involves total thyroidectomy or excision of the recurrent lesion and associated lymph nodes. However, success rates vary significantly, ranging between 19% and 67% (5, 6). Reoperation is challenging due to scar tissue from previous surgeries, increasing the risk of complications such as recurrent laryngeal nerve injury (0 - 3% in primary surgery but up to 12% during reoperation), parathyroid damage, and neck nerve dysfunction (7).

To reduce these complications, minimally invasive techniques have been developed (8). Radiofrequency ablation (RFA) utilizes thermal energy to ablate tumors in situ, proving effective for locoregional recurrences (9). Ultrasound (US)-guided percutaneous laser ablation (PLA) applies laser energy to destroy tissue, effectively treating low-risk subcentimeter PTC lesions (10). Percutaneous ethanol injection (PEI) involves the injection of ethanol into thyroid cysts, adenomas, and cervical lymph node metastases (11), offering a potentially effective approach for treating PTC lesions while minimizing the surgical risks associated with reoperation.

A 2015 meta-analysis found that PEI had higher failure and recurrence rates compared to reoperation; however, more recent studies indicate that PEI is an effective, safe, cost-efficient, and well-tolerated option for patients who are either ineligible for or choose not to undergo surgery (12-14). Despite these promising findings, there are notable limitations in the current research on PEI for treating recurrent PTC. To date, no prospective randomized controlled trials have compared PEI directly with surgery for managing persistent or recurrent PTC metastasis. Few studies directly contrast PEI and surgery (14), and reports on PEI for neck nodal metastasis remain limited by small sample sizes and short follow-up durations, with the longest follow-up period reported being 65 months (15).

## 2. Objectives

This study aims to report the mid-term outcomes of PEI in patients with a history of PTC surgery and local recurrence in cervical lymph nodes, marking the first investigation of this kind conducted in Iran.

## 3. Methods

### 3.1. Selection of Patients for Ethanol Ablation

Between October 2017 and September 2021, patients with a history of PTC who had undergone thyroidectomy, lymph node dissection, and radioactive iodine therapy (RAIT), and who presented with cervical lymph node recurrence, were considered for this study at Taleghani Hospital in Tehran. Patients who were either unable or unwilling to undergo reoperation were eligible for PEI treatment following specific inclusion and exclusion criteria. A total of 20 patients were enrolled, with diagnoses confirmed by ultrasonography-guided fine-needle aspiration (FNA) before the procedure. These patients were followed up until May 2022, with a median follow-up duration of 21.4 months.

Participants were eligible for inclusion if they met all of the following criteria: (1) patients with local recurrence of PTC in cervical lymph nodes who were poor surgical candidates (either at high risk for general anesthesia due to a medical condition or for surgery because of prior repeated neck dissections) and/or preferred to avoid additional surgery; (2) presence of carcinoma cells in FNA specimens and/or elevated FNA-thyroglobulin (FNA-Tg) levels; (3) no radiological evidence of malignancy outside of the neck region; (4) history of previous treatment with radioactive iodine (131I); and (5) lymph nodes not located near major vessels to minimize the risk of ethanol intravasation, as determined by a radiologist.

**Figure 1.**
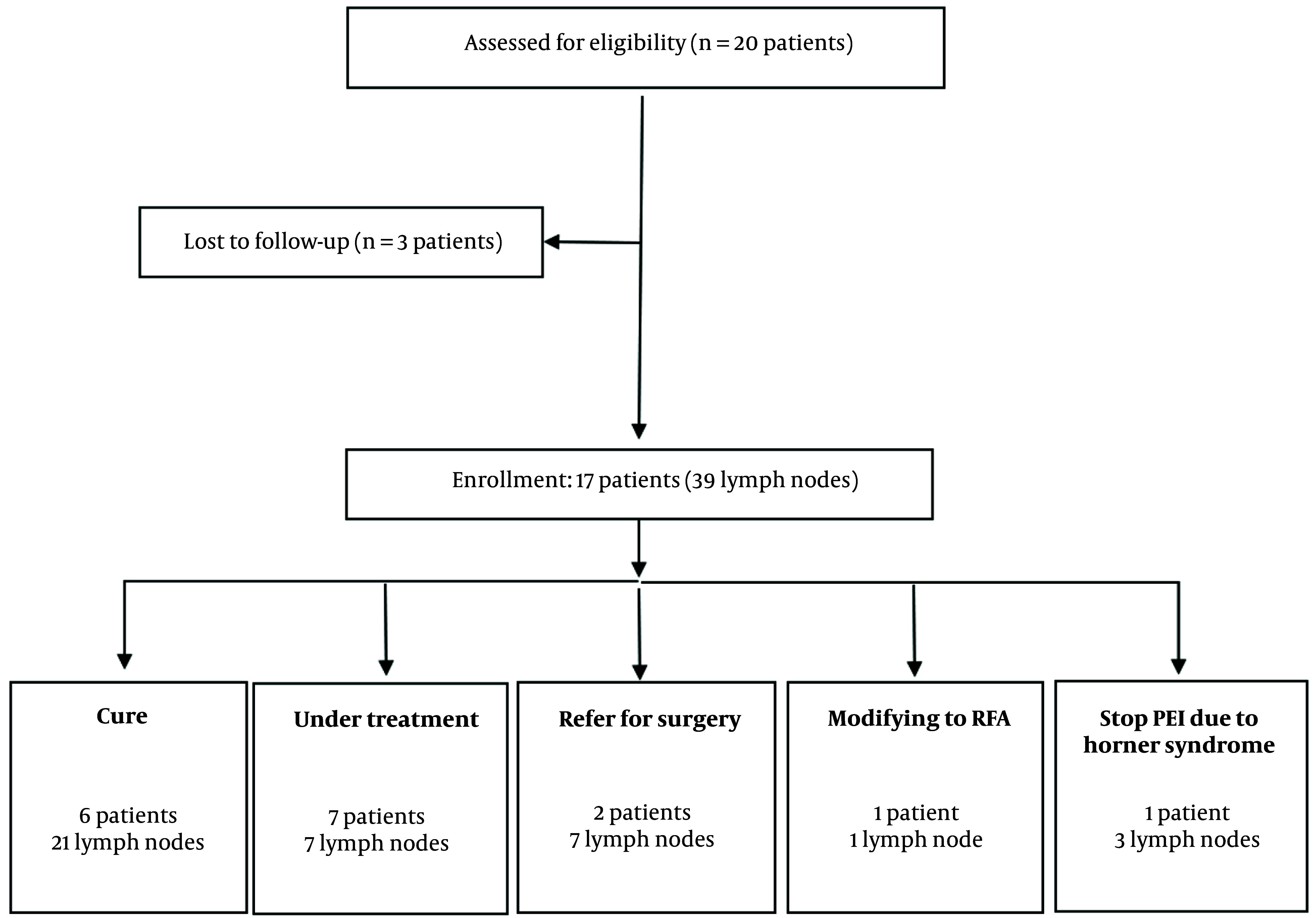
Cure: Of the 21 cases classified as cured, 12 demonstrated complete disappearance, while 9 exhibited a reduction in size by more than 50%. PEI, percutaneous ethanol injection; RFA, radio frequency ablation.

Out of the twenty patients initially included in this study, three were excluded due to lack of follow-up after their initial PEI session. Consequently, 39 lesions were treated in the remaining 17 patients. During the follow-up period, four patients (involving 11 lymph nodes) did not complete the full PEI course: Two patients discontinued PEI and opted for surgery due to lymph node progression, one patient developed Horner syndrome, and another chose to switch to RFA. The remaining 28 neck lymph nodes were treated with PEI as a monotherapy (Figure 1). 

Clinical information, including tumor-node-metastasis (TNM) staging based on the American Joint Committee on Cancer (AJCC) guidelines at the time of the primary diagnosis, was retrospectively recorded (16). All 17 patients had localized disease with no distant metastasis. The initial TNM stages were stage I in 9 patients and stage II in 8 patients. Among these, 25 cases of local PTC recurrence in cervical lymph nodes were detected primarily, while 14 additional tumor foci in the neck were identified during follow-up, distinct from the primary lesions. Of the 39 lymph nodes treated, 10 were located within the surgical bed, and the remaining 29 were situated outside the surgical bed.

### 3.2. Details of the Ethanol Ablation Procedure

In this study, all follow-up ultrasounds and ethanol injections were performed by a skilled radiologist with approximately 15 years of experience in thyroid procedures. Informed consent was obtained from all patients prior to each procedure. Routine measurements of coagulation times and platelet counts were not conducted, and no patients received premedication. Post-procedure, outpatient care was provided, with patients observed in the radiology recovery room for 1 to 2 hours under continuous vital sign monitoring before discharge.

Ultrasound using a 12 - 5 MHz transducer was performed every three months to monitor lymph nodes. If lymphadenopathy was detected, FNA under US guidance was performed, and ethanol injection was administered if metastasis was confirmed. An experienced radiologist evaluated the lymph nodes, using color Doppler to assess vascularity before treatment. Fine-needle aspiration material was collected with a 25- or 27-gauge needle and examined by a cytopathologist before PEI, with lymph nodes classified according to the AJCC staging system (16).

The PEI procedure followed the protocol described by Lewis et al. using a 25-gauge needle and 99.5% ethanol (17). A freehand US-guided puncture technique was used, injecting 0.1 to 1 mL of ethanol into the periphery of the lymph node while withdrawing the needle. Larger nodes required repositioning of the needle for multiple injections to ensure complete ablation. Color Doppler imaging was used to monitor vascularization, with additional injections administered to vascularized areas. The volume of ethanol used and any complications were recorded, with additional ethanol administered via a second needle if required.

### 3.3. Follow-up Protocol

Ultrasonography was conducted every three months to evaluate the efficacy of PEI. The lymph nodes’ diameters were measured in three dimensions: Longitudinal, transverse, and anteroposterior, and tumor vascularity was examined. The pre-treatment and post-treatment volumes of each lymph node were calculated using a standard formula based on the anteroposterior (a), transverse (b), and longitudinal (c) measurements.

The criteria for successful PEI treatment were: (1) complete disappearance of the lymph nodes, or (2) a reduction in lymph node volume of at least 50% without detectable vascularization. Lymph nodes that met these criteria were subsequently monitored per the follow-up schedule. For lymph nodes with persistent intralesional signals or signs of enlargement, additional PEI sessions were administered to achieve satisfactory ablation. The number of PEI sessions per lymph node, as well as the volume of ethanol used per session, were recorded.

### 3.4. Statistical Analysis

Data normality was assessed using the Kolmogorov-Smirnov test and by examining histograms. Study results were reported as medians with minimum-maximum values for quantitative variables, and as frequencies with percentages for categorical variables. Lymph node volume was calculated using the formula: Volume = 4/3 × π × a/2 × b/2 × c/2 and the percentage volume change of each lymph node was calculated as: Vchange % (percent volume decrease) = (V_1_ - V_2_)/V_1_) × 100; V_1_ is the pre-treatment volume, and V_2_ is the post-treatment volume. Percutaneous ethanol injection efficacy was determined by calculating the percentage reduction in lymph node volume, and success was defined by complete or partial resolution of nodules.

## 4. Results

A total of 17 patients (7 male, 10 female) with a history of PTC surgery and locally recurrent cervical lymph nodes were monitored for a median duration of 21.4 months (range: 4.1 - 37.9). The median age at initial diagnosis was 34 years (range: 20 - 57), and the median age at the time of PEI treatment was 38 years (range: 23 - 61). All patients had previously undergone total thyroidectomy with central lymph node dissection, followed by RAIT with a cumulative dose of 200 mCi (range: 150 - 600 mCi). Additionally, 7 patients had undergone modified radical neck dissection.

Prior to PEI, median thyroglobulin (Tg) and thyroid-stimulating hormone (TSH) levels were 0.2 ng/mL (range: 0.04 - 4.5) and 0.05 mIU/mL (range: 0.02 - 0.14), respectively. Post-PEI, these values changed to 0.5 ng/mL (range: 0.04 - 0.78) for Tg and 0.2 mIU/mL (range: 0.08 - 3.2) for TSH. Anti-thyroglobulin (anti-Tg) levels were also measured in all patients; however, Tg and anti-Tg data for two patients with persistently elevated anti-Tg levels were excluded from the analysis (Table 1). 

**Table 1.** Patient Characteristics ^a, b^

Variables	Values; N = 17
**Age at diagnosis**	34 (20 - 57)
**Age at treatment**	38 (23 - 61)
**Sex, (male) **	7 (41)
**Median follow-up duration, (mo) **	21.4 (4.1 - 37.9)
**Previous operation**	
Total thyroidectomy and central node dissection	17 (100)
Modified radical neck dissection	7 (41)
**Total RAI dosage, mCi**	200 (150 - 600)
**First-TSH, mIU/mL**	0.05 (0.02 - 0.14)
**last-TSH, mIU/mL**	0.2 (0.08 - 3.2)
**First-Tg, ng/mL**	0.2 (0.04 - 4.5)
**last-Tg, ng/mL (on levothyroxine)**	0.5 (0.04 - 0.78)

Abbreviations: RAI: radioactive Iodine; TSH: thyroid Stimulating Hormone; Tg: thyroglobulin.

^a^ Data are expressed as n (%) or median (minimum-maximum).

^b^ First-TSH and First-Tg refer to their serum levels before the initial ethanol Injection, while the last-TSH and last-Tg represent their serum levels during the final follow-up visit.

During the follow-up period, 39 recurrent lesions were identified across the 17 patients. Of these, 25 lesions were initially detected, and 14 were subsequently identified as new lesions. Among the lymph nodes, 23 were located in the lateral zones, while 16 were in the central zone. In 31 lesions, the mean number of ethanol injections was three or fewer, while the remaining lesions required more than three injections. The median initial volume of the lesions decreased from 0.12 mm³ (range: 0.06 - 0.34 mm³) to 0.03 mm³ (range: 0.0 - 0.14 mm³), reflecting a 72.6% (range: 20.0 - 100.0%) volume reduction and an absolute volume change of 0.06 mm³ (range: -1.54 - 0.7 mm³) (Table 2). 

**Table 2.** Lesion Characteristics ^a, b, c, d, e^

Variables	Values; N = 39
**Number of lesions**	
Initial	25 (64)
Follow-up	14 (36)
**Site of percutaneous ethanol injection **	
Central	16 (41)
Lateral	23 (59)
**Number of percutaneous ethanol injection**	
≤ 3	31 (80)
> 3	8 (20)
**V **_**1**_ **, mm** ^**3**^	0.12 (0.06 - 0.34)
**V **_**2**_ **, mm** ^**3**^	0.03 (0.0 - 0.14)
**V **_**change**_	72.6 (20.0 - 100.0)
**Absolute V **_**change**_ **, mm** ^**3**^	0.06 (- 1.54 - 0.7)

^a^ Data are expressed as n (%) or median (minimum-maximum).

^b^ V_1_: Pretreatment volume; V_2_: Posttreatment volume.

^c^ V (mm^3^) = 4/3 × π × a/2 × b/2 × c/2.

^d^ V_change_ (%) = (V_1_ - V_2_)/V_1 _× 100 (V_1_: Pretreatment volume, V_2_: Post treatment volume)

^e^ Absolute V _change_ (mm^3^) = V _1_ - V _2_.

Of the 21 lymph nodes that were successfully treated in 6 patients, 12 nodes completely disappeared, while 9 showed a volume reduction of more than 50% without any visible vascularization. In the non-cured group, 11 lesions were identified: Seven of these lesions proceeded to surgery, including one patient with multiple new metastatic neck lymph nodes (6 lesions) and another whose lesion volume progressed from 0.7 to 2.2 cm after two PEI sessions. One patient opted to switch to RFA. In another case, treatment was halted due to the development of Horner syndrome. A total of 7 lymph nodes remain under ongoing treatment (Figure 1). 

Regarding the safety of alcohol injection, most patients tolerated the treatment well, experiencing only mild pain at the injection site and reporting no other significant complaints. However, one patient developed symptoms of Horner's syndrome following the fifth alcohol injection session. This patient required multiple sessions due to recurrent lesions.

## 5. Discussion

Our study aimed to evaluate the mid-term outcomes of PEI in patients with recurrent PTC in cervical lymph nodes who were either poor surgical candidates or preferred not to undergo further surgery. Our findings indicate that PEI is an effective, safe, and well-tolerated minimally invasive treatment option for selected PTC patients with local recurrence in cervical lymph nodes. Of the 39 lymph nodes treated, 21 met the criteria for successful treatment, while treatment for 7 lymph nodes is ongoing. In response to an increase in lymph node size or the emergence of complications, surgery was required for seven lymph nodes in two patients.

Percutaneous ethanol injection is a minimally invasive procedure involving the injection of ethanol into thyroid cysts or nodules under US guidance, promoting scar tissue formation and reducing nodule size. Percutaneous ethanol injection has been used for over two decades to treat solitary hot, toxic, and cold thyroid nodules and is considered the first-line treatment for thyroid cystic nodules (3). Percutaneous ethanol injection is also recommended for managing PTC recurrence in cervical lymph nodes, particularly for patients who are unsuitable for surgery or wish to avoid further operations (18). Ethanol induces cell death by causing cellular dehydration, protein denaturation, coagulative necrosis, and blood vessel thrombosis (19), resulting in stable regression of the treated cystic lesion.

Cervical lymph node metastases in PTC may necessitate additional treatment beyond surgery and RAIT (20). One alternative treatment under study is PEI for metastatic lymph nodes. Since Lewis et al. (17) first reported favorable outcomes from ethanol injection for recurrent neck lymph node disease in 2002, multiple studies have demonstrated satisfactory results. Previous short-term studies on PEI, with follow-up periods ranging from 18 to 65 months, have reported success rates between 79% and 100% (17, 21). In our study, 39 lymph nodes were treated, and after a median follow-up of 21.4 months (range 4.1 - 37.9), 21 lymph nodes (54%) met the criteria for cure, while 7 lymph nodes (18%) remain under observation.

The safety of PEI is essential for its clinical viability. In our study, one patient developed Horner’s syndrome, which ultimately led to surgical intervention. No other significant complications, such as vocal cord palsy or tracheal injury, were observed, consistent with previous findings on PEI’s safety (12, 13, 21). Prior research has identified mild, self-limited pain and discomfort at the injection site as the most common side effect, likely due to minimal ethanol leakage into surrounding tissue (12). Percutaneous ethanol injection can become more challenging with an increased number of involved lymph nodes, leading to longer procedure times and greater patient discomfort. Larger lesions present technical difficulties and often require multiple sessions for complete ablation. In our study, 8 lymph nodes (20%) required more than three PEI sessions. Thus, PEI demands careful patient selection and skilled operators to optimize outcomes.

Notable trials with extended follow-up periods in the United States, Norway, and Korea have shown that PEI produces acceptable outcomes with minimal side effects for treating PTC lymph node recurrence (12, 13, 22). For instance, Frich et al. (22) re-evaluated 44 patients who had received PEI for metastatic lymph nodes from PTC, with a median follow-up of 124 months. Of the 67 lymph nodes initially treated, 97% met response criteria by the end of the earlier study (11). At the latest follow-up, 81% (54/67) maintained a durable response, while 19% (13/67) experienced recurrence. Although no randomized trials have directly compared PEI with surgery or other procedures, guidelines continue to favor surgery due to limited long-term follow-up data (3). Nonetheless, the low cost and minimal side effects of PEI suggest it could be a viable treatment alternative for PTC lymph node recurrence.

This study represents the first evaluation of ethanol injection for PTC recurrence in Iran. All procedures were conducted by a single radiologist with over 15 years of experience, enhancing the reliability of the findings. However, the study has limitations, including a relatively small sample size and the absence of a control group, which limits comparisons with other treatments such as reoperation or RFA. While the follow-up period of 21.4 months was longer than that of several similar studies, it still did not allow for an assessment of the long-term effects of PEI on recurrence rates. Given that PTC metastases can occur up to 47 years post-treatment (23), with a mean recurrence time of 5.1 years (24), larger studies with extended follow-up are necessary to confirm the long-term efficacy and safety of PEI for PTC patients.

In conclusion, our study demonstrated that PEI is an effective and safe minimally invasive treatment option for PTC recurrence in cervical lymph nodes for selected patients who are either ineligible for surgery or decline reoperation.

## Data Availability

The dataset presented in the study is available on request from the corresponding author during submission or after publication.
